# Supplementary figures and images for: Genomic epidemiology reveals antibiotic resistance transfer and polyclonal dissemination of Acinetobacter baumannii in a Paraguayan hospital
Source: Antimicrob Agents Chemother. 2025 Jul 8;69(8):e00077-25. doi: 10.1128/aac.00077-25 (PMC12326978; doi:10.1128/aac.00077-25)

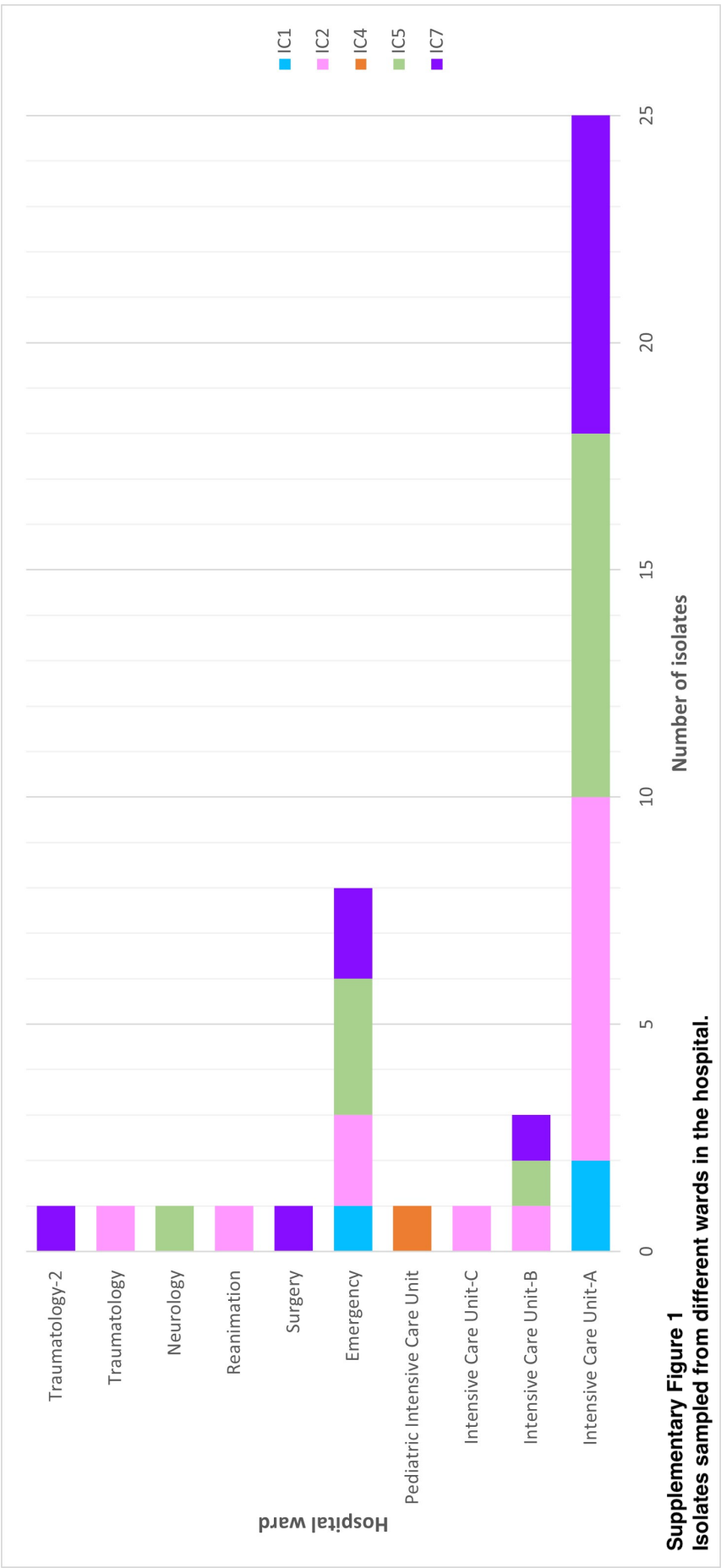

**Supplementary Figure 1**  
Isolates sampled from different wards in the hospital.

Supplement: Fig. S1 — Isolates sampled from different wards in the hospital. [file aac.00077-25-s0001.pdf]
