# Supplementary material for: Genomic epidemiology reveals antibiotic resistance transfer and polyclonal dissemination of Acinetobacter baumannii in a Paraguayan hospital
Source: Antimicrob Agents Chemother. 2025 Jul 8;69(8):e00077-25. doi: 10.1128/aac.00077-25 (PMC12326978; doi:10.1128/aac.00077-25)
Supplement: Fig. S2 — Genomic context of armA, and aph(3) and aph(6) in the IC2 isolates. [file aac.00077-25-s0002.pdf]

A) armA genomic context

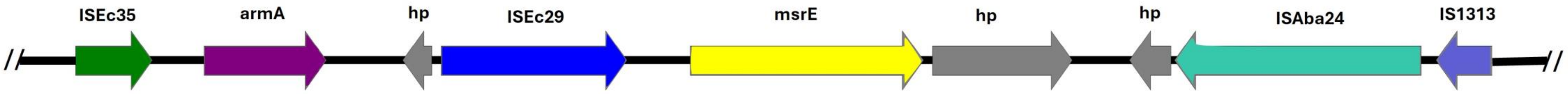

12 isolates have this configuration

B) aph(3) and aph(6) genomic context

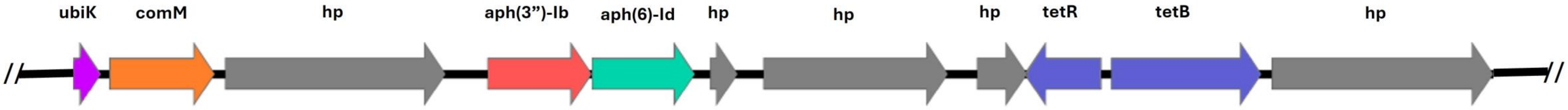

All the IC2 isolates have this arrangement

Supplementary Figure 2
Genomic context of armA, and AMEs in the IC2 isolates
