# Supplementary material for: Genomic epidemiology reveals antibiotic resistance transfer and polyclonal dissemination of Acinetobacter baumannii in a Paraguayan hospital
Source: Antimicrob Agents Chemother. 2025 Jul 8;69(8):e00077-25. doi: 10.1128/aac.00077-25 (PMC12326978; doi:10.1128/aac.00077-25)
Supplement: Fig. S3 — Plasmid profiles of 17 Paraguayan isolates. [file aac.00077-25-s0003.pdf]

Supplementary Figure 3.
Plasmid profiles of 17 Paraguayan isolates.

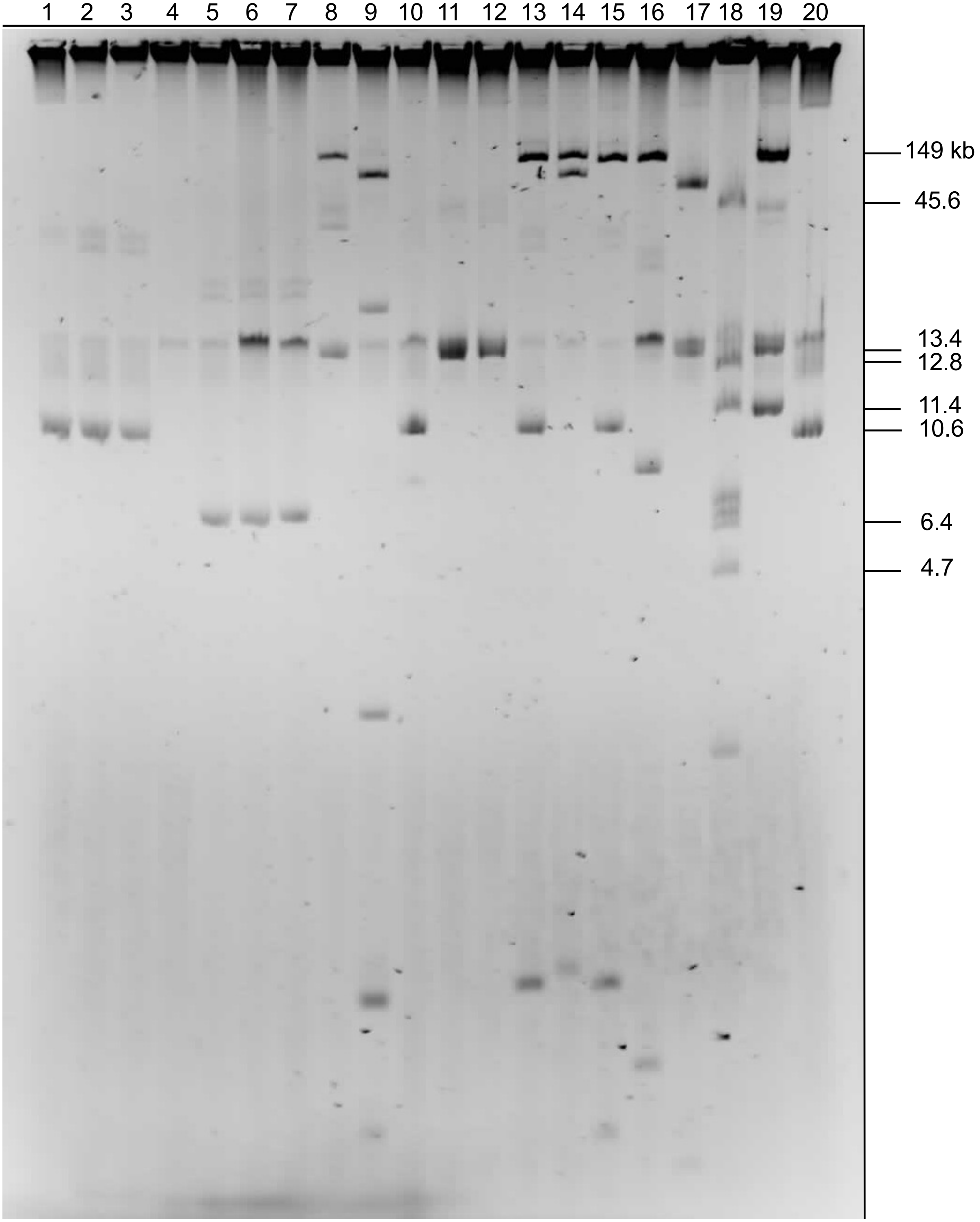

Lane 1, AbHTMGP-5566. Lane 2, AbHTMGP-388. Lane 3, AbHTMGP-1125. Lane 4, AbHTMGP-4911. Lane 5, AbHTMGP-4696. Lane 6, AbHTMGP-4701. Lane 7, AbHTMGP-4888. Lane 8, AbHTMGP-3145. Lane 9, AbHTMGP- 4173. Lane 10, AbHTMGP-2026. Lane 11, AbHTMGP-2957. Lane 12, AbHTMGP-2693. Lane 13, AbHTMGP-4330. Lane 14, AbHTMGP-863. Lane 15, AbHTMGP-4890. Lane 16, AbHTMGP-3916. Lane 17, AbHTMGP-2689. Lane 18, A. haemolyticus AN54. Lane 19, A. baumannii ATCC17978. Line 20, E. coli DH5a/pTR102.
